# Access to specialist community alcohol treatment in England, and the relationship with alcohol-related hospital admissions: qualitative study of service users, service providers and service commissioners

**DOI:** 10.1192/bjo.2020.80

**Published:** 2020-08-25

**Authors:** Emmert Roberts, Miriam Hillyard, Matthew Hotopf, Stephen Parkin, Colin Drummond

**Affiliations:** National Addiction Centre and the Department of Psychological Medicine, Institute of Psychiatry, Psychology and Neuroscience, Kings College London and the South London and the Maudsley NHS Foundation Trust, UK; National Addiction Centre, Institute of Psychiatry, Psychology and Neuroscience, Kings College London, UK; Department of Psychological Medicine, Institute of Psychiatry, Psychology and Neuroscience, Kings College London; and the South London and the Maudsley NHS Foundation Trust, UK; National Addiction Centre, Institute of Psychiatry, Psychology and Neuroscience, Kings College London, UK; National Addiction Centre, Institute of Psychiatry, Psychology and Neuroscience, Kings College London; and the South London and the Maudsley NHS Foundation Trust, UK

**Keywords:** Alcohol disorders, service users, qualitative research, service provision, hospitalisation

## Abstract

**Background:**

Since 2012 England has seen year-on-year reductions in people accessing specialist community alcohol treatment, and year-on-year increases in alcohol-related hospital admissions.

**Aims:**

We examined perceived barriers to accessing specialist treatment, and perceived reasons behind hospital admission increases.

**Method:**

We conducted focus groups (*n* = 4) with service users and semi-structured interviews (*n* = 16) with service providers and service commissioners at four specialist community alcohol services in England, which experience either high or low rates of alcohol dependence prevalence and treatment access. Themes and subthemes were generated deductively drawing upon Rhodes’ risk environment thesis. Data were organised using the framework approach.

**Results:**

Data reveal a treatment sector profoundly affected at all levels by changes implemented in the Health and Social Care Act (HSCA) 2012. Substantial barriers to access exist, even in services with high access rates. Concerns regarding funding cuts and recommissioning processes are at the forefront of providers’ and commissioners’ minds. The lack of cohesion between community and hospital alcohol services, where hospital services exist, has potentially created an environment enabling the reduced numbers of people accessing specialist treatment.

**Conclusions:**

Our study reveals a treatment sector struggling with a multitude of problems; these pervade despite enaction of the HSCA, and are present at the national, service provider and individual service level. Although we acknowledge the problems are varied and multifaceted, their existence is echoed by the united voices of service users, service providers and service commissioners.

## Background

In 2012 the Health and Social Care Act (HSCA) gained royal assent under the UK coalition government. This cemented in legislation the transfer of specialist drug and alcohol service commissioning responsibilities from the National Health Service (NHS) and placed this statutory duty solely within the remit of local authorities.^1^ Since 2012 specialist alcohol treatment has seen year-on-year reductions in real-term funding,^2^ major changes in the alcohol treatment provider landscape, and England has faced a period of ‘austerity’ in which economic conditions have been created by government measures to reduce public expenditure. Currently approximately 65% of specialist community alcohol services are provided by third sector or private sector agencies, compared with prior to the Act's passage when over 90% of services were provided by the NHS.^2–4^

## Scale of the problem

The number of alcohol-related hospital admissions has been concurrently rising in England over the past decade. In 2018/19 there were an estimated 1.3 million hospital admissions, an increase of 8% compared with 2017/18, and of 14% compared with 2008.^5^ In 2015 the regulatory body responsible for public health, Public Health England, reported high levels of variability across the 152 local authorities in England in both the adult prevalence of alcohol dependence (ranging from 0.64% in Wokingham to 3.85% in Blackpool), and the percentage of this population accessing specialist community alcohol treatment (ranging from 41.47% in Solihull to 6.93% in Sandwell).^6^ These estimates are in the context of a national decline in the number of people accessing specialist community treatment for alcohol problems, this having fallen by 19% from a peak of 65 110 in 2013/14 to 52 383 in 2016/17.^3^

Previous qualitative research has demonstrated that service users find it difficult to navigate through specialist alcohol services in England and that perceived fragmentation of treatment journeys has had a negative impact on service-user engagement,^7,8^ but to our knowledge no qualitative research has been conducted within the specialist community alcohol treatment sector since the passage of the HSCA to examine what are considered to be the barriers that prevent service users accessing specialist community alcohol services, nor what are perceived to be the reasons behind increases in alcohol-related hospital admissions, and any associated dynamics with specialist services.

## Aims

We aimed to investigate the perceptions of service users, service providers and service commissioners within four specialist community alcohol services in England that experience either high or low rates of alcohol dependence prevalence and high or low rates of specialist treatment access.

## Method

This study is reported according to the consolidated criteria for reporting qualitative research,^9^ the completed checklist can be found in supplementary Table 1; available at https://doi.org/10.1192/bjo.2020.80. The authors assert that all procedures contributing to this work comply with the ethical standards of the relevant national and institutional committees on human experimentation and with the Helsinki Declaration of 1975, as revised in 2008. All procedures involving human patients were approved by the King's College London Psychiatry, Nursing and Midwifery Research Ethics Subcommittee (Reference HR-18/19-5360). The study benefited throughout from discussion with the South London and the Maudsley Biomedical Research Centre Service User and Carer Advisory Group, which provided input into the study design, participant information leaflets and amendments to the development and phraseology of all topic guides. Written informed consent was obtained from all participants.

### Recruitment of local authority commissioned specialist community alcohol services

Using publicly available data on alcohol dependence prevalence and the percentage of people with alcohol dependence accessing specialist community alcohol treatment,^6^ we purposively initially approached, via email, the four local authority commissioned specialist community alcohol services that comprised the area in England with the combined:

- highest access and highest prevalence (HAHP);
- highest access and lowest prevalence (HALP);
- lowest access and highest prevalence (LAHP); and
- the lowest access and lowest prevalence (LALP).

**Fig. 1:**
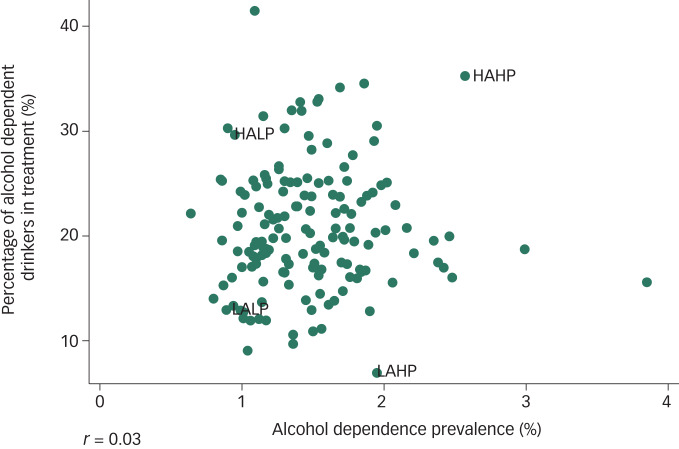
The relationship between the proportion of alcohol dependent drinkers accessing treatment (%) and the prevalence of alcohol dependence (%) across the 152 local authorities in England.

Where gatekeeper approval was granted these areas, and their corresponding specialist community alcohol services were recruited, and where this was refused the next area with the highest combined score in their respective access and prevalence category was approached. This continued until one area in each of the four categories outlined above had been recruited. The study team had no relationships with any recruited services or study participants prior to study commencement. A scatter diagram depicting the relationship between alcohol dependence prevalence and the percentage of people with alcohol dependence accessing treatment across the 152 local authorities in England can be found in Fig. 1. This indicates a lack of correlation between population need and population uptake (*r* = 0.03).

*r*, Pearson correlation coefficient; HAHP, high access high prevalence; HALP, high access low prevalence; LAHP, low access high prevalence; LALP, low access low prevalence.

### Recruitment of participants

In each of the four services we aimed to recruit service users, service providers and the service commissioner to provide perspectives from three key alcohol service stakeholder groups. We aimed to recruit a sample of 4–6 service user participants to take part in a 1-hour focus group. The decision to utilise focus groups was considered acceptable given that group formats are commonly deployed in alcohol treatment settings, and this format was thought to be more appropriate than one-on-one interviews in order to encourage discussion and directly observe commonalities and differences between service user participants.^10,11^ To be included a service user was required to be older than 18 years, have a diagnosis of alcohol dependence and be receiving treatment from the specialist alcohol service in question. They were each provided with a reciprocal payment of £20 in cash for their time and effort,^12^ this was approved by the Research Ethics Committee and not considered to be coercive. Individuals with alcohol dependence who were not currently accessing specialist community alcohol treatment were not recruited for this study.

In addition to the service users in each of the four services we recruited the service commissioner responsible for commissioning the service, and three healthcare professionals (HCPs) who worked within the service providing care for people with alcohol dependence. These participants were invited to take part in a 30–45 min semi-structured interview. Interviews were considered pragmatic as these participants often have large demands on their time, and may choose not to disclose perceived sensitive information in front of their colleagues. Professionals were identified through snowball sampling within services, and could be from a medical, nursing, psychology or recovery worker background.

### Data collection

E.R. conducted all interviews and focus groups face to face at either the service team base or local authority offices, only the interviewer and study participants were present. E.R. is a male practising academic psychiatrist with over 8 years’ experience of facilitating groups and conducting interviews with people with substance misuse. All participants were provided with participant information leaflets that highlighted the reasons for conducting the research, and the aims of the researcher (see supplementary Figs 1 and 2). Written informed consent was obtained from all participants. Interviews and focus groups were based on topic guides (see supplementary Figs 3, 4 and 5), which were developed and pilot tested in April 2017. During interviews and focus groups field notes of observations, contextual information and reflections were recorded. No repeat interviews were conducted.

### Analysis

Data analysis drew upon Rhodes’ risk environment thesis that argues that certain interactions between individuals and various contextual factors at different environmental levels (macro, meso and micro) can function to increase or decrease risks (and thus produce or reduce harm).^13^ As such a deductive approach to analysis was employed in which data were simultaneously organised using the framework approach,^11,14–16^ this systematically organises and categorises data to identify emerging themes and subthemes. The key stages are familiarisation, identifying a coding index, indexing, charting and mapping and interpretation.^11^

Interviews and focus groups were audio recorded and transcribed verbatim. All transcripts were checked for accuracy, and prior to coding an initial coding frame was developed to reflect the risk environment theory, the topic guide and notes taken during the familiarisation stage. This coding frame was constructed to allow researchers to ascribe data to themes within three hierarchical levels. These levels (macro, meso and micro) reflected the level of service to which the participant was referring and were consistent with the risk environment model. These were defined *a priori* as the national level (macro), the level of the service provider (for example Change Grow Live, Cranstoun, Turning Point) (meso) and the level of the individual specialist alcohol service (for example HALP, HAHP) (micro).

Two members of the research team (E.R. and M.Hi.) initially coded a quarter of all transcripts independently line by line, allowing for additional inductive themes to emerge from the data. After this preliminary analysis, the emergent coding frame was discussed between all authors to obtain different reflective perspectives on the data. Where questions were raised, the transcripts were re-examined, and the coding frame adjusted to reflect new insights and understandings of the data. Once a coding frame had been agreed, all remaining transcripts were independently coded by the aforementioned two researchers and the coding frame iteratively adjusted for consistency and rigour at predefined intervals. The final coding frame used during deductive framework analysis can be found in supplementary Table 2. Verbatim quotes were used to illustrate themes and subthemes, and the results reported considering the differences elicited between the level of service (macro, meso or micro), the type of participant (service user, service commissioner or HCP) and the characteristics of the service in question (HAHP, HALP, LAHP or LALP). The analysis was supported by NVivo software version 12.

## Results

Recruitment and data collection took place between April 2018 and July 2019 (see supplementary Fig 6 for recruitment flow diagram). The characteristics of recruited participants are shown in Table 1.

**Table 1:** Characteristics of participants

Profession/participant ID	Gender	Age, year	Alcohol Use Disorder Identification Test (AUDIT) Score^17^	Severity of Alcohol Dependence Questionnaire (SADQ) Score^18^
*High access low prevalence*				
Interviews				
Service commissioner	Man	46	N/A	N/A
Recovery worker (HCP1)	Woman	37	N/A	N/A
Recovery worker (HCP2)	Man	39	N/A	N/A
Recovery worker (HCP3)	Man	44	N/A	N/A
Focus group				
Service user 1	Woman	54	21	16
Service user 2	Woman	50	24	36
Service user 3	Woman	47	37	15
Service user 4	Woman	45	27	45
Service user 5	Man	38	33	23
*Low access low prevalence*				
Interview				
Service commissioner	Woman	53	N/A	N/A
Recovery worker (HCP1)	Woman	42	N/A	N/A
Nurse (HCP2)	Woman	29	N/A	N/A
Recovery worker (HCP3)	Man	37	N/A	N/A
Focus group				
Service user 1	Woman	44	26	20
Service user 2	Woman	56	20	8
Service user 3	Woman	45	25	16
Service user 4	Man	45	40	34
Service user 5	Man	42	40	33
Service user 6	Man	53	31	29
*High access high prevalence*				
Interviews				
Service commissioner	Man	29	N/A	N/A
Recovery worker (HCP1)	Man	30	N/A	N/A
Recovery worker (HCP2)	Woman	52	N/A	N/A
Recovery worker (HCP3)	Man	36	N/A	N/A
Focus group				
Service user 1	Woman	47	36	52
Service user 2	Man	40	26	25
Service user 3	Woman	52	40	56
Service user 4	Woman	57	32	31
*Low access high prevalence*				
Interview				
Service commissioner	Woman	40	N/A	N/A
Recovery worker (HCP1)	Woman	44	N/A	N/A
Recovery worker (HCP2)	Woman	26	N/A	N/A
Recovery worker (HCP3)	Woman	38	N/A	N/A
Focus group				
Service user 1	Man	48	28	26
Service user 2	Man	35	19	8
Service user 3	Woman	48	36	49

HCP, healthcare professional.

Three major themes relating to the research questions posed emerged from the data. These are summarised, along with subthemes and an example of a relevant quotation in Table 2.

**Table 2:** Summary of themes and subthemes

Theme	Subtheme (and example of relevant quote)
1. Who does and does not receive specialist alcohol treatment	1.1 Characteristics of those receiving treatment ‘Full range of people. I was really surprised that when I started… because you do just imagine a particular group of people when you first start, but I've worked with social workers, teachers, nurses, really successful people and then people that are homeless.’ (LAHP – health care professional 1)
	1.2 Characteristics of those not receiving treatment ‘I think there is this kind of hidden middle-class cohort.’ (HAHP – service commissioner)
	1.3 Priority groups ‘If there is clients who are a particular risk, so domestic violence or sort of safeguarding issues and that kind of thing, we tend to fast track them.’ (HALP – health care professional 2)
	1.4 Excluded groups ‘I think somebody has been banned, I think, but that was just for unacceptable behaviour and I think it was pretty extreme.’ (LAHP – health care professional 2)
	1.5 Complex needs ‘I think they're very good at including people with complex care needs.’ (LALP – service commissioner)
	1.6 Working with mental health teams ‘The biggest problem we have is with mental health. The brick wall between mental health and substance misuse. Yes. It's very much… Well, it's just frustrating, really frustrating for everybody.’ (LALP – health care professional 2)
2. Barriers and facilitators of access to specialist alcohol treatment services	2.1 Barriers to access specialist alcohol services
	2.1.1 Stigma associated with specialist alcohol services ‘When my GP referred me originally to here, he said, oh perhaps you shouldn't actually come there. And I went, what do you mean? He said, you're a bit of a lady. I said, what do you mean by that? He said, they'll upset you there.’ (LALP –service user 1)
	2.1.2 Stigma to individuals ‘They're not the sort of things that you go out and advertise to other people, to say, look at me, I'm a binge drinker, yay!’ (HALP – service commissioner)
	2.1.3 Transport ‘Because the area's so big and actually for some people it's two buses to get to here.’ (LAHP – health care professional 3)
	2.1.4 Location of service ‘I think its location doesn't work for a lot of people.’ (HALP – service commissioner)
	2.1.5 Co-location with drug services ‘So things like the age old thing of I don't want to sit here with a smackhead, to use the proverbial, and you know, I only drink I don't want nothing to do with it, there's a lot of that you know, whatever your substance is, there's inter-substance warfare going on.’ (HAHP – health care professional 3)
	2.1.6 Personal barriers ‘It was all that fear based stuff that… and it was, I can do this on my own if I just… I can control it.’ (HAHP – service user 3)
	2.1.7 Other ‘Well, I don't think this place is very well known about to start with.’ (LAHP – service user 3)
	2.2 Facilitators to access specialist alcohol services ‘We do do outreach venues, which again that is the element that makes it easier for people.’ (LAHP – health care professional 3)
	2.3 Innovation and creativity ‘I think it's just – but to be creative like that, we needed to find a venue that was free, because we didn't have the money.’ (LALP – health care professional 1)
	2.4 Funding cuts ‘Cuts, Cuts, Cuts! God knows what's going to happen in terms of future funding and the public health grant, but where we're going, we can't keep on doing what we are doing.’ (LAHP – service commissioner)
	2.5 Recommissioning ‘I mean it just seems sort of like every 18 months or something catastrophic happens and somebody else is involved and nobody really knows what's going on, it's not very, there's no clarity over who's going to be the service provider and even if they say we've got it for two years, I'm always cynical about that and I think everybody is.’ (HAHP – health care professional 3)
	2.6 Marketing and promotion of specialist alcohol service ‘I think we need to be out there more, promoting our service, which I think the problem with that is by the time we get out there and get ourselves known, everything's changed.’ (LAHP – health care professional 2)
3. Hospital admissions	3.1 Experiences of hospitalisation due to alcohol ‘I broke my arm in May. Ended up in A&E and they kept asking me how did you break your arm? I said I fell off my bike. Obviously a lie. I just fell over because I was pissed.’ (HALP – service user 5)
	3.2 Characteristics of hospital attendees ‘Sometimes people see them [the local hospital] as, “Well, if they get me into hospital then they'll give me something to detox me. I've got no money for alcohol so I'm going to end up going through withdrawal”.’ (HAHP – health care professional 2)
	3.3 Trends in hospitalisation ‘I think partially it is the fact that you've got an older generation of people that are now suffering with the effects of drinking for a long period of time and not getting support.’ (HALP – health care professional 1)
	3.4 Relationship of services with hospital ‘We used to go in every day, we used to have a worker go in every day, go through the wards, but that's exactly the same as I've said about the posters and that; Some of the staff didn't even know who we were and you'd go in there every day, still don't know who you are.’ (LAHP – health care professional 2)

HAHP, high access high prevalence; HALP, high access low prevalence; LAHP, low access low prevalence; LALP, low access low prevalence.

### Theme one: who does and does not receive specialist alcohol treatment

The characteristics of those receiving treatment were reported similarly across all services, and by all participants. It should be noted all services participating in the research were adult services and therefore were commissioned to only treat service users above the age of 18. Service users accessing services were reported to span all socioeconomic strata, however, several commissioners thought that despite an ‘open door policy’ certain groups were less likely to attend their service; these included concerns that those with mobility issues, and ‘middle class’ drinkers were at risk of reduced access, the latter largely because of stigma.

‘Middle class ladies or men at home, don't want to come in through the door.’ (LALP – HCP3)

All professionals and the commissioner at the LAHP service were concerned that people with severe alcohol dependence were at greater risk of harm than previously as they were not accessing treatment in the numbers they had since 2012.

‘We would see very dependent drinkers. What we're starting to see is more lower-level, increasing and higher risk drinking profiles coming through the service, and fewer dependent drinkers.’ (LAHP – service commissioner)

Although all services reported some ad-hoc prioritisation or ‘fast-tracking’ of individuals with specific safeguarding needs, only the LALP service providers reported specific protocols for prioritisation of pregnant women, and those deemed at risk of suicide. No one reported prioritisation of individuals based on pattern of drinking behaviour or any comorbidity or complex need. Only one instance of an individual being formally excluded from a service was reported because of extreme behavioural problems.

‘I think somebody has been banned, I think, but that was just for unacceptable behaviour and I think it was pretty extreme.’ (LAHP – HCP2)

No one reported that people with complex needs were excluded from the service, although some professionals pointed out the capacity to conduct home visits was no longer feasible, and as such people who were housebound, although still able to access telephone support, were not able to make use of the full suite of treatment modalities.

Zero positive interactions with mental health services were reported and frequent examples were cited of service users being at an increased risk because of their inability to access local mental health services because of alcohol misuse. There was a reported lack of confidence and training to deal with mental health problems within specialist alcohol services, statements such as ‘we are not a crisis team’ were common among professionals. There were reported ‘brick walls’ between mental health services and alcohol services, with some commissioners reporting that they felt mental health teams often used alcohol misuse as a blanket reason for non-acceptance of referrals to their service.

‘The biggest problem we have is with mental health. The brick wall between mental health and substance misuse. Yes. It's very much… Well, it's just frustrating, really frustrating for everybody.’ (LALP – HCP3)

### Theme two: barriers and facilitators of access to specialist alcohol treatment services

At all levels, barriers to accessing specialist alcohol treatment could be broken down into factors related to the service itself and factors related to individuals. Service-related factors included the stigma attached to specialist alcohol services, the geographical location of the service, the building in which the service was provided and whether the alcohol service was co-located with drug services.

All service users and professionals reported experiences of stigma associated with their service, often from within the healthcare sector. Multiple accounts of local general practitioners (GPs) or hospital services not wanting to refer people as it was deemed ‘not your kind of place’ were reported, particularly in areas of low access.

‘When my GP referred me originally to here, he said, oh perhaps you shouldn't actually come there. And I went, what do you mean? He said, you're a bit of a lady. I said, what do you mean by that? He said, they'll upset you there.’ (LALP – service user 1)

The physical location of the service was frequently cited as a problem; a single hub point of access within a large local authority area was described as inconvenient for people not located close to the hub. The presence of the service in a shopping centre in the HAHP service meant some individuals could not access the service as they had been banned from accessing the shopping centre. Individuals reported having to take two buses to access the LAHP service, which was both costly and time consuming, and once there, the area where the service is situated was described as ‘dodgy’ and ‘unsafe’, leading to people being reluctant to visit. Co-location with drug services was reported as an initial barrier in three services, several service users stated this had been an initial barrier but once ‘though the door’ this was less of an issue. Other service-related barriers discussed included lack of awareness of services existence, the expectation that people would have to participate in group settings, and the lack of services available outside working hours.

‘Because the area's so big and actually for some people it's two buses to get to here.’ (LAHP – HCP3)

With regard to individual-related barriers, the stigma, shame and guilt associated with alcohol misuse was reported widely by all participants. In addition, a number of personal barriers were discussed including a lack of motivation to change, failure to accept that drinking was having a negative impact on life and an unwillingness to accept the need for formal support; factors deemed likely to prolong ongoing harm from alcohol. Some service users and one professional expressed that their allegiance to mutual aid organisations had, in some cases, led to their not wishing to attend specialist alcohol services or to usher service users away from treatment within the statutory sector.

There were a number of reported facilitators to accessing services – outreach and in-reach capability most often highlighted by professionals. Those services with outreach hubs, and in-reach into hospitals described an ability to locate potential service users and engage them prior to formal enrolment into the specialist alcohol service. Two services had invested in specific outreach initiatives to identify and engage people with severe alcohol dependence not currently in contact with treatment services.

‘Well, I don't think this place is very well known about to start with.’ (LAHP – service user 3)

Marketing and promotion of services were consistently seen as both important and often lacking by service users. In the LAHP service the commissioner reported that marketing campaigns in local GP services, and on social media had widened the franchise of those accessing treatment. Several professionals cited workload as a barrier to innovation, and wished they could accomplish more creative ways of marketing the service. While the LALP professionals had held stalls at local community events, the financial and opportunity cost associated with publicity and marketing were cited as limiting factors in being able to do this kind of work.

‘I think we need to be out there more, promoting our service, which I think the problem with that is by the time we get out there and get ourselves known, everything's changed’ (LAHP – HCP2)

The two areas discussed more than any other were service recommissioning and funding cuts, both being reported by all participants as having had a significant negative impact since enactment of the HSCA. Across all levels, service recommissioning processes were felt to be poorly communicated and contributory to low staff morale and burnout, this mediated a reduction in access via a desire for staff to limit what were seen as ‘ever expanding caseloads’.

Professionals often had turbulent working environments with limited expectations of job security, were unsure when contracts were due for renewal and what would happen in the event of a tender being awarded to a different provider. Service users seemed mostly oblivious to the process other than confusion as to why names and locations of services have changed, but professionals and commissioners regularly stated they felt this lack of awareness on the part of service users was largely because of professionals shielding them from any deleterious consequences of the recommissioning process. A result of recommissioning was seen by service users as representing the risk of a change in their allocated keyworker, and thus potential loss of a therapeutic relationship.

‘I think that's the case with all the changes that have been made, after the initial kind of anxiety and sort of uncertainty and sort of, you know everything's a bit up in the air.’ (HALP – HCP3)

All participants spontaneously reported the perceived major funding cuts, particularly in the past 5 years. Participants perceived these as having resulted in, among other aspects, higher keyworker case-loads, an increased reliance on a volunteer workforce, an inability to provide high-quality services and a reduction in trained staffing levels. In the LALP service a publicity campaign had successfully resulted in a freeze on further cuts within the current calendar year, however, the level of overall funding was still significantly lower than it had been 5 years previously. All commissioners expressed concern over the future of public health spending by central government, and thought along the lines of the ‘the worst is yet to come’ with the risk of further cuts anticipated. Some stated that potential providers had dropped out because of the limited availability of adequate funding resulting in an increased number of tendering cycles. Service users experienced a reduction in available treatment modalities, increased waiting times to see prescribers, a fear that their keyworkers were going to be out of a job and worries that reduction in funding would mean they may return to drinking without the current level of support they were receiving.

Most service users blamed central government for funding reductions, whereas professionals blamed both central government and local authorities. No one described a future where funding would be restored to previous levels, and the vast majority of participants regarded ongoing funding cuts with a degree of inevitability.

### Theme three: hospital admissions

All service users and professionals reported experiences of service users being admitted to hospital because of alcohol; these were varied with some service users describing it as a pivotal moment in their treatment journey that led to them being linked with specialist community services, and ultimately receiving appropriate support. Others reported negative experiences including being inadequately detoxified and hurriedly discharged, being told ‘you shouldn't come here [Accident and Emergency Department]’ with alcohol-related problems.

‘We're not going to detox you, you've been discharged.’ (HALP – service user 1)

The most frequently cited reasons for hospital admissions were because of wholly attributable alcohol conditions, including alcohol withdrawal syndrome, alcoholic liver disease and injuries sustained secondary to intoxication.

Service users perceived austerity, and an increased availability and affordability of alcohol as major factors influencing the increasing number of alcohol-related hospital admissions. Professionals from some services thought recent ‘cuts, cuts, cuts’ to specialist alcohol services may have also played a role. Several commissioners stated that poor dialogue and communication between acute hospitals and specialist services may be responsible. They reported that, because of differing hospital and specialist service catchment areas, there was often confusion on the part of hospitals as to which service the service user in question ‘belongs to’. The HAHP service providers reported an ‘excellent’ alcohol liaison team within their local hospital that would conduct screening and signposting to their service, although this team had been decommissioned and cut in the month prior to our interviews. Many professionals stated that information sharing with hospitals was difficult in particular for third-sector-provided services. Often hospital professionals did not ‘believe’ the specialist alcohol service had consent to access hospital records; in one instance this lack of information sharing was felt to have led to a serious untoward incident.

‘We're phoning them up and try to say get any details when we've got consent to share, won't tell us anything, won't tell us anything at all…. The primary concern is the individual, that's the kernel of it all, isn't it, we're just the husk, wanting to help… once we didn't know someone was suicidal and no one said anything.’ (HALP – HCP3)

## Discussion

### Main findings

This study explored service user, provider and commissioner views on access to specialist community alcohol services and associated alcohol-related hospital admission in England. The themes elicited paint a varied picture, but overall they reveal a treatment sector that has been profoundly affected at all levels, macro (national level), meso (service-provider level) and micro (individual specialist alcohol service level), by the changes implemented in the HSCA 2012. There are substantial barriers to accessing specialist community alcohol treatment, even in services with high access rates, and concerns regarding funding cuts and the recommissioning process are at the forefront of the minds of providers and commissioners. As alcohol-related harm, as evidenced by alcohol-related hospital admission, continues to rise year-on-year, viewed through the lens of Rhodes’ risk environment, the lack of cohesion between community and hospital alcohol services, where hospital services exist, has potentially enabled an environment that has led to reduced numbers of people in specialist alcohol treatment, and increased the risk of preventing useful interventions or appropriate signposting being delivered during hospital admission episodes.^19^

### Issues with knowledge, visibility and accessing of services

Despite purposively sampling areas of high and low access the experiences described by all participants reflected a strong desire to treat anyone and everyone who accesses the service. The personal barriers to accessing specialist treatment described are similar to those elicited from international samples,^20,21^ and although some barriers reported have been highlighted by previous governmental investigations into treatment access rates^3^ knowledge of services’ existence by both service users and within the healthcare sector has always previously generally been taken as read. The lack of knowledge and visibility of services has not created an enabling macro environment for service users to access specialist care, and increased the risk of non-engagement with specialist treatment. The discussion of the need for active marketing and promotion has not historically been factored into service budgets, nor to our knowledge has there been any previous evidence suggesting it may be necessary in England to directly influence alcohol-related harm.

### Limitations

The study aimed to recruit a broad sample of services and participants. Although it was planned to include a variety of professional staff to provide broad insight into the research topic, largely only recovery workers took part, none of whom represented senior staff within the service in which they worked. As such findings may not reflect the diversity of professions within specialist alcohol staff, nor the range of seniority.

In addition, in the LAHP service we were only able to recruit three service user participants to take part in the focus group, which may have limited the reported diversity of service user experiences within that service. The research may have been limited by the sampling strategy to those services that agreed to take part, and uptake may have been higher had services been incentivised to participate. Likewise, participating service users were by design currently receiving specialist alcohol treatment, and thus differing barriers and experiences are likely in those individuals whom have not accessed or not been able to access services. Although experiences described by participants encompass a variety of perceptions, they cannot be representative of all national services.

The role of the interviewer, and any preconceptions they may have, can lead to the introduction of bias within qualitative studies in both the way questions are put to participants and how data is coded. It should be noted that the lead researcher and interviewer is a practising psychiatrist within the NHS, and any potential bias was attempted to be mitigated by co-production of topic guides with the service user and carer advisory group, which are available in supplementary Figs 3, 4 and 5, the use of the structured framework approach, double coding with a second researcher and triangulation of all findings with the full research team. However, some of their experiences are highlighted in the wider international research literature suggesting that although some problems may be unique to England, there are also issues, such as co-location of drug services,^22^ and the need for an appropriate physical space that are pertinent across the international alcohol treatment landscape. The choice of focus group methodology with this population may have led to difficulties in some members expressing their views or being overshadowed.^23^

### Integration with mental health service support

At the macro, meso and micro levels the lack of joined up working, and barriers to accessing mental health service support was abundant. Within England the structure of statutory services is delineated by diagnosis, such that people experiencing problems with alcohol and/or drug use are referred to treatment within drug and alcohol services, and those with serious mental illness (such as paranoid schizophrenia, bipolar affective disorder) or common mental disorders (such as depressive and anxiety disorders) are treated within secondary mental health services, provided they cannot be managed in primary care. Medications for alcohol use disorders are typically initiated in drug and alcohol services and continued in primary care. Discussion of the consequences of siloed mental health and substance misuse treatment systems is also not unique to the UK,^24^ nor indeed to this era. However, our study adds to the evidence base that the issues outlined with mental health treatment not only pervade alcohol treatment systems but are currently acutely felt in England and potentially worsened by the changes implemented in the HSCA that have led to fewer alcohol services within the NHS compared with their mental health team counterparts.

It should be noted these views of mental health service integration may not be reflective of all geographies or services, and although the reflections on mental health services here are overwhelmingly negative, caution should be advised in interpretation because of variation in practice and provision nationally and over time. Our results suggest the current macro treatment environment is not one that can adequately support the needs of a high proportion of its service users, and there is thus a need for specific policies at national, regional and local levels to promote mental health and substance use service integration and facilitate a discussion of how both treatment systems can function to reduce risk, reduce alcohol harm and be of overall benefit to their service users. This is particularly concerning because of the reportedly observed reductions in access from severe dependent drinkers, whom are most likely to have comorbidity and complex needs.

### Role of alcohol care teams

Hospital admission has the potential to be not merely a venue for crisis detoxification, but a pivotal point in a service user's alcohol treatment journey. As part of the NHS Long Term Plan,^25^ the UK government has committed to developing alcohol care teams in the 25% of hospitals in England that have the greatest burden of alcohol-related admissions. Although this intent accords in principle with our findings, given the fact that specialist care teams exist nationally for many other conditions (such as diabetes mellitus), an ambition for alcohol care teams in every hospital would appear warranted to address this observed risk in a treatment sector environment that recognises the need for holistic working across the spectrum of health and social care.

### Implications

Although the study is unable to separate the effects of the HSCA and ‘austerity’ as both were applied during this time period, the enactment of the HSCA made austerity-based funding cuts to drug and alcohol services more possible because of the removal of ring-fenced budgets for drug and alcohol provision within an NHS commissioning structure, as this was relatively protected from austerity measures compared with local authority budgets. In addition, the prevalence of alcohol misuse in England has been largely static since the passage of the HSCA and the coinciding period of austerity, suggesting reported changes in service-user drinking profiles are unlikely to be largely driven national changes in drinking patterns.^22^ The localist approach to specialist alcohol treatment delivery was intended to provide areas with the necessary resources, and the flexibility to take into account local needs. Our study reveals a specialist alcohol treatment sector that is struggling with a multitude of ongoing problems. These issues pervade despite the enaction of the HSCA, and are present at the national, service provider and individual service levels. While we acknowledge the problems are varied and multifaceted, the potential increased risk of alcohol-related harm is echoed by the united voices of service users, service providers and service commissioners.

## Data Availability

Authors have full and ongoing access to all study data.

## Supplementary material

For supplementary material accompanying this paper visit http://dx.doi.org/10.1192/bjo.2020.80. click here to view supplementary material
