# Supplementary material for: Access to specialist community alcohol treatment in England, and the relationship with alcohol-related hospital admissions: qualitative study of service users, service providers and service commissioners
Source: BJPsych Open. 2020 Aug 25;6(5):e94. doi: 10.1192/bjo.2020.80 (PMC7488322; doi:10.1192/bjo.2020.80)
Supplement: Supplementary file 1 [file bjosup.zip › S2056472420000800sup001.docx]

**Online Supplementary Material**

Contents

Figures

S1. Service user participant information leaflet

S2. Healthcare professional and service commissioner participant information leaflet

S3. Topic guide for service user participants

S4. Topic guide for healthcare professional participants

S5. Topic guide for service commissioner participants

S6. Recruitment flow diagram

Tables

S1. COREQ checklist

S2. Final coding index

Figures

Figure S1: Service user participant information leaflet


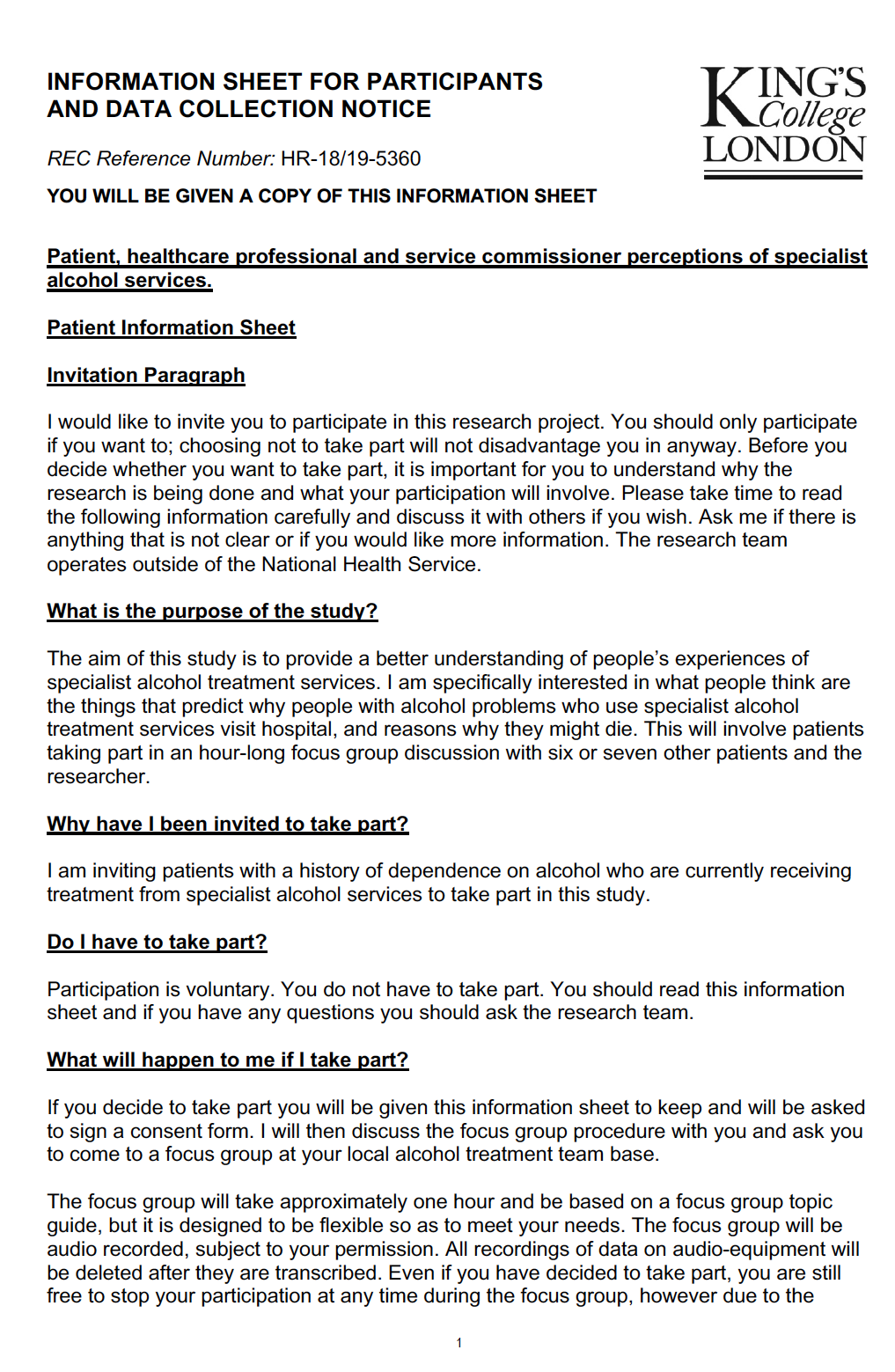


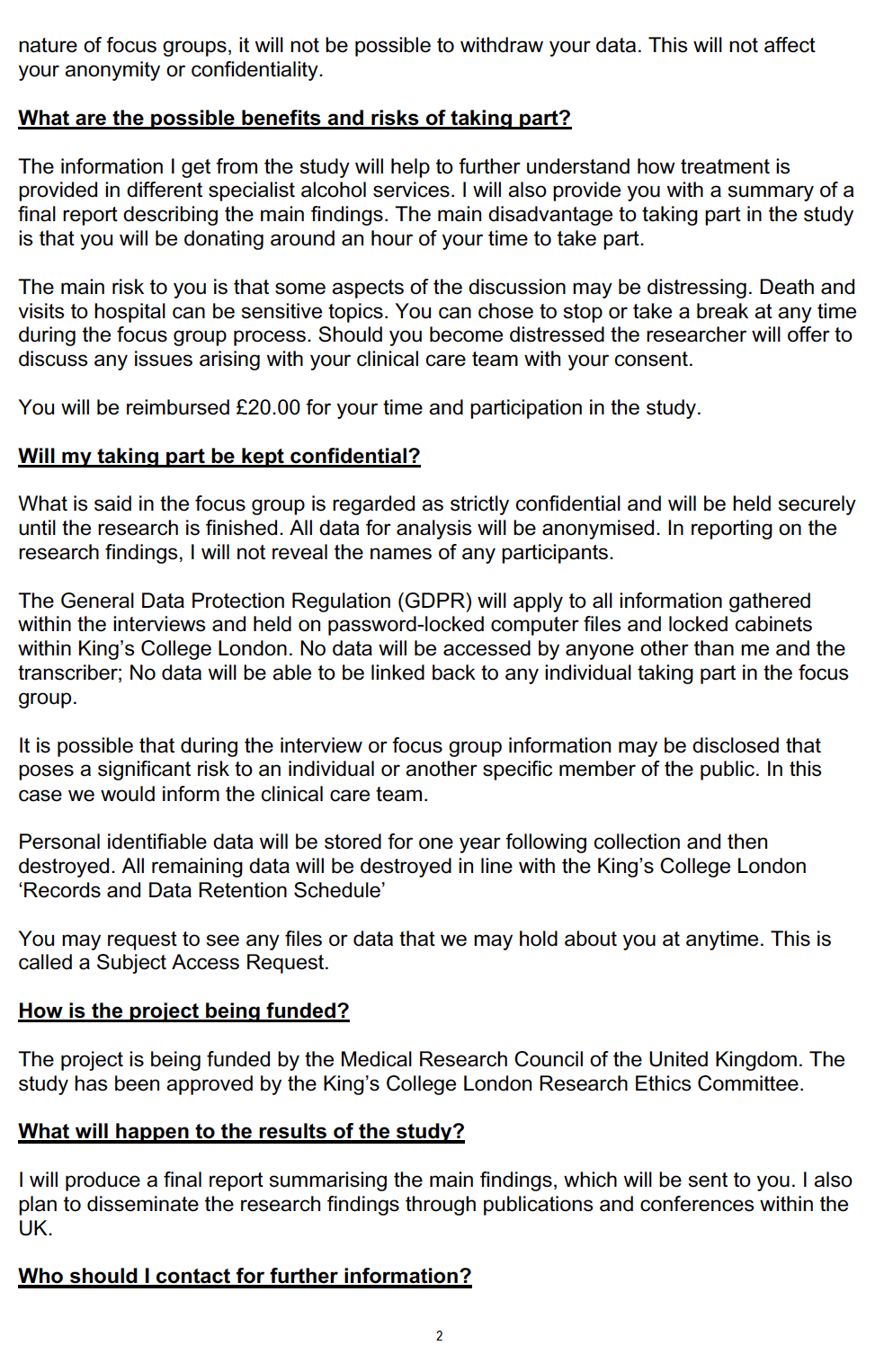


Figure S2: Healthcare professional and service commissioner participant information leaflet


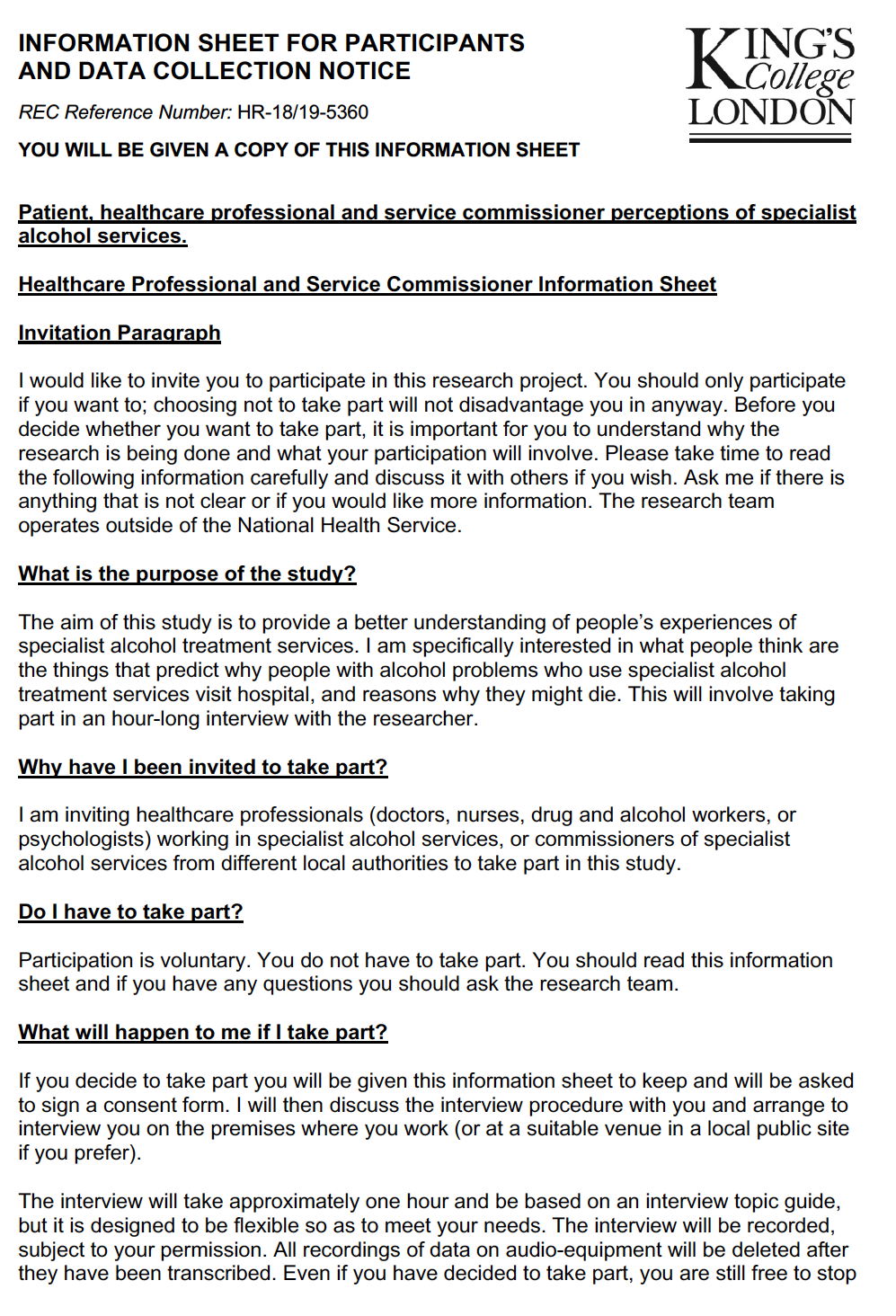


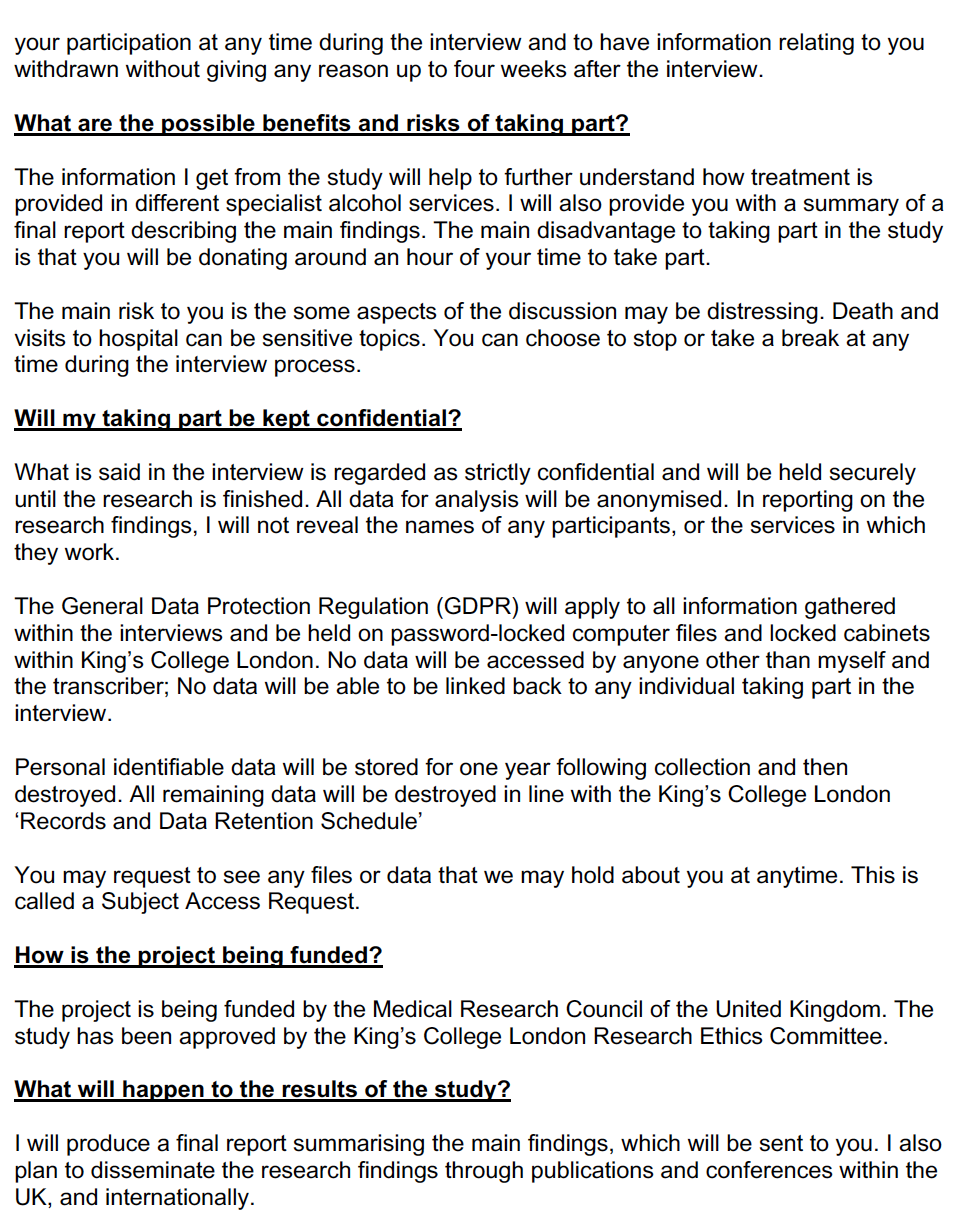


Figure S3: Topic guide for service user participants

**Focus group topic guide: Service Users**

**Introduction to Individual Participants**

*Thank you for agreeing to take part in this research and for meeting me today.*

Review the Participant Information Sheet and ensure the participant fully understands the information and answer any questions they may have.

Go through each point in the consent form and be clear about confidentiality and anonymity.

Ask to confirm whether the participant is content for the focus group to be recorded.

Ensure written consent is obtained from each participant before commencing the focus group.

**Introduction to Focus Group**

*This focus group is about your experiences of your treatment by the community alcohol service. It is an opportunity for you to talk about how you’re thinking and feeling at present, about the care and treatment you received.*

*I do not work for this team and will not feed any of your responses directly back to them. The study will be written up as a report and published but any quotes used will be completely anonymous and you will not be identified anywhere in the report.*

*I am a researcher working for King’s College London working to improve the quality of care for patients in alcohol services. I’m interested in your experience and your attitude to the treatment you received in the alcohol service. We would like your honest, candid views, positive, negative and neutral. Nothing you say in this interview will influence your personal care you receive in the alcohol service, if there are any aspects that arise during the discussion that suggest a significant risk to yourself or others I will notify the clinicians in the team.*

*There are no right or wrong answers. So, on the whole the focus group will be guided by the things you want to talk about. If we start to discuss anything you’re not comfortable discussing, you do not have to take part. Please let me know and we can stop or take a break at any time you would like, and you are free to leave the focus group if you wish to do so.*

**Overall experience of alcohol services**

*Q1 Thinking about every aspect of the specialist alcohol service, what is your overall impression of the treatment of your alcohol problem by the specialist alcohol service? What have been the key issues for you?*

Probes: Which parts of the service had the biggest impact on you? Why? How did the clinical team affect your experience? What did having specialist treatment mean to you? What were the problems or challenges in receiving treatment?

**Treatment**

*Q1 You were invited to take part in this research because you have undergone specialist treatment for problems with alcohol. Would you mind telling me a little about what kinds of treatment you’ve received?*

Probes: Medication? Psychology? Detoxification? Peer Support?

**Access**

*Q2 What kind of people are treated at the service?*

*Q3 Are there any barriers preventing people from accessing the specialist alcohol service?*

Probes: Stigma?, Lack of awareness of service? Transport?

*Q5 What things make it harder or easier for people to access the service? Has this changed at all?*

Probes: Different providers can have different methods of ‘getting people through the door’

*Q6 Are some people prioritised for treatment over others? Is there anyone who is excluded from the service?*

*Q7 What about people with complex needs?*

Probes: Physical or mental health problems; Homeless; Housing needs

*Q8 Do you work with anyone else or other teams? Do they work together or communicate with the specialist service?*

Probes: Joint working arrangements with primary care, probation, secondary mental health services, housing etc.

**Hospitalisation**

*Q9 Do you have any experiences of needing to go to hospital for problems relating to alcohol? / Do you think you’ve ever needed to go to hospital due to problems relating to alcohol?*

Probes: Any alcohol related injuries, Any visits to A+E? Spent time in ITU or critical care?

*Q10 What do you think about going to hospital for alcohol problems? How would you describe the links between the specialist alcohol service and hospital? How would you describe the interactions between hospitals and the specialist alcohol service?*

Probes: Communication between service and hospital? Any promotion or restriction of hospital admissions?

*Q11 The number of people going to hospital due to an alcohol related problem has been rising over the last few years, why do you think that might be?*

**Mortality**

*Q11 I realise this can be a sensitive subject can I ask what are your thoughts about dying due to problems related to alcohol?*

Probes: Do you have any experiences of people you know who have died due to alcohol related problems?

*Q12 What do you think about the reasons people die due to alcohol problems? Do you talk about death or dying in specialist alcohol services?*

*Q13 The number of alcohol related deaths has been rising over the last few years, why do you think that might be?*

**Conclusions**

Summarise the main topics discussed and check with the participants that they are a good representation of the interview.

**Closure**

*Is there anything that we haven’t covered? Would you like to add something that we haven’t had a chance to talk about yet?*

*What do you feel was the most important topic we discussed today?*

*Do you have any questions?*

*Thank you very much for taking part in this research*

Figure S4: Topic guide for healthcare professional participants

**Interview topic guide: Healthcare Professionals**

**Introduction**

*Thank you for agreeing to take part in this research and for meeting me today.*

Review the Participant Information Sheet and ensure the participant fully understands the information and answer any questions they may have.

Go through each point in the consent form and be clear about confidentiality and anonymity.

Ask to confirm whether the participant is content for the interview to be recorded.

Ensure written consent is obtained before commencing the interview.

*This interview is about your experiences of the specialist alcohol service you work in. It is an opportunity for you to talk about how you’re thinking and feeling at present.*

*The study will be written up as a report and published but any quotes used will be completely anonymous and you will not be identified anywhere in the report.*

*I am interested in your experience and your attitude towards the specialist alcohol service. We would like your honest, candid views, positive, negative and neutral.*

*There are no right or wrong answers. So, on the whole the interview will be guided by the things you want to talk about. If we start to discuss anything you’re not comfortable discussing, let me know and we’ll move on to something different. We can stop or take a break at any time you would like.*

**Participant background**

*Q1. Could you start by telling me a little about your role in the specialist alcohol service?*

*Q1. How long have you been in your role? Do you have any previous experience of working in specialist alcohol services?*

**Changes in service provision**

*Q2. What changes have you experienced or observed in the provider of specialist alcohol services. If so in what way, if any, has that changed the service and your role?*

*Q3*. *How has your role changed, if at all? How do you feel about that?*

**Experience of commissioning changes on the service**

*Q5. What has been your experience of the commissioning process on the service overall?*

Probes: What have been the challenges and strong points?

*Q6. How has it impacted on the clinical team?*

**Experience of commissioning process on patient care**

*Q7. How has the commissioning process impacted on patient care and patient wellbeing?*

Probes: Which aspects do you think have had the most positive impact on patients? Which aspects have been most problematic? What have been the barriers? Are there any specific cases that would illustrate your points? How have patient consultations changed? To what extent are we able to draw general conclusions?

**Access and priority patient groups**

*Q8. Who are the kinds of patients you see?*

Probes: Priorities for certain kinds of patient? Anyone excluded?

*Q9. Are some people prioritised for treatment over others? Is there anyone who is excluded from the service?*

*Q10. What about people with complex needs?*

Probes: Physical or mental health problems; Homeless; Housing needs

*Q11. Are there any barriers preventing people from accessing the specialist alcohol service?*

Probes: Stigma?, Lack of awareness of service? Transport?

*Q11. What things make it harder or easier for people to access the service? Has this changed at all?*

Probes: Different providers can have different methods of ‘getting people through the door’

*Q12. Do you have any joint working arrangements? Are there any issues with other teams?*

Probes: Joint working arrangements with primary care, probation, secondary mental health services, housing etc.

**Hospitalisation**

*Q13. Who in your experience are the kind of patients that go to hospital due to problems related to alcohol?*

Probes: Frequent attendees to A+E? Detoxification? Comorbidities?

*Q15. The number of people going to hospital due to an alcohol related problem has been rising over the last few years, why do you think that might be?*

**Mortality**

*Q16. Who in your experience are the kind of patients who die due to problems related to alcohol?*

Probes: Age? Homeless?

*Q17 The number of people dying hospital due to an alcohol related problem has been rising over the last few years, why do you think that might be?*

**Conclusions**

Summarise the main topics discussed and check with the participant that they are a good representation of the interview.

**Closure**

*Is there anything that we haven’t covered? Would you like to add something that we haven’t had a chance to talk about yet? What do you feel was the most important topic we discussed today?*

*Do you have any questions?*

*Thank you very much for taking part in this research*

Figure S5: Topic guide for service commissioner participants

**Interview topic guide: Service Commissioners**

**Introduction**

*Thank you for agreeing to take part in this research and for meeting me today.*

Review the Participant Information Sheet and ensure the participant fully understands the information and answer any questions they may have.

Go through each point in the consent form and be clear about confidentiality and anonymity.

Ask to confirm whether the participant is content for the interview to be recorded.

Ensure written consent is obtained before commencing the interview.

*This interview is about your experiences of the specialist alcohol service you commission. It is an opportunity for you to talk about how you’re thinking and feeling at present.*

*The study will be written up as a report and published but any quotes used will be completely anonymous and you will not be identified anywhere in the report.*

*I am interested in your experience and your attitude towards the specialist alcohol service you commission. We would like your honest, candid views, positive, negative and neutral.*

*There are no right or wrong answers. So, on the whole the interview will be guided by the things you want to talk about. If we start to discuss anything you’re not comfortable discussing, let me know and we’ll move on to something different. We can stop or take a break at any time you would like.*

**Participant background**

*Q1. Could you start by telling me a little about your role in commissioning the specialist alcohol service?*

*Q1. How long have you been in your role? Do you have any previous experience of working in specialist alcohol services?*

**Changes in service provision**

*Q2. Have there been any changes in provider of specialist alcohol services. If so in what way, if any, has that changed the service and your role?*

Probes: How has your role changed? How do you feel about that?

**Experience of commissioning changes on the service**

*Q3. What has been your experience of the commissioning process for the service overall?*

Probes: What have been the challenges and strong points? How has it impacted on the team?

**Experience of commissioning process on patient care**

*Q5. How has the commissioning process impacted on patient care and patient wellbeing?*

Probes: Which aspects do you think have had the most positive impact on patients? Which aspects have been most problematic? What have been the barriers? Are there any specific cases that would illustrate your points? How have patient consultations changed? To what extent are we able to draw general conclusions?

*Q6. What factors have affected the level of service provision?*

**Access and priority patient groups**

*Q6. Who are the kinds of patients seen?*

Probes: Priorities for certain kinds of patient? Anyone excluded?

*Q7. Are some people prioritised for treatment over others? Is there anyone who is excluded from the service?*

*Q8. What about people with complex needs?*

Probes: Physical or mental health problems; Homeless; Housing needs

*Q9. Are there any barriers preventing people from accessing the specialist alcohol service?*

Probes: Stigma?, Lack of awareness of service? Transport?

*Q10. What things make it harder or easier for people to access the service? Has this changed at all?*

Probes: Different providers can have different methods of ‘getting people through the door’

**Hospitalisation**

*Q11. Do you think the commissioning process has had any impact on people attending hospital due to problems with alcohol?*

Probes: Frequent attendees to A+E? Detoxification? Comorbidities?

*Q11. The number of people going to hospital due to an alcohol related problem has been rising over the last few years, why do you think that might be?*

**Mortality**

*Q12. Do you think the commissioning process has had any impact on people dying due to problems with alcohol?*

Probes: Age? Homeless?

*Q13. The number of people dying due to an alcohol related problem has been rising over the last few years, why do you think that might be?*

**Conclusions**

Summarise the main topics discussed and check with the participant that they are a good representation of the interview.

**Closure**

*Is there anything that we haven’t covered? Would you like to add something that we haven’t had a chance to talk about yet? What do you feel was the most important topic we discussed today?*

*Do you have any questions?*

*Thank you very much for taking part in this research.*

Figure S6: Recruitment flow diagram

Local authority commissioned specialist alcohol services

Contacted via email and follow up telephone call (N=6)

Declined (N=1)

(Prospective LAHP)

No response (N=1)

(Prospective HALP)

Access gained

(N=4)

Access not gained

(N=2)

HAHP

HALP

LAHP

LALP

Focus Group (N=3; 1M/2F)

Professionals (N=2; 2RW)

Service Commissioner (N=1;M)

Focus Group (N=5; 1M/3F)

Professionals (N=2; 2RW)

Focus Group (N=2; 1M/1F)

Service Commissioner (N=1;M)

Focus Group (N=6; 2M/2F)

Professionals (N=2; 2RW)

Service Commissioner (N=1;F)

Service Commissioner (N=1;F)

Professionals (N=2; 1RW/1N)

Tables

Table S1: COREQ checklist

| **No** | **Item** | **Guide questions/description** |
| --- | --- | --- |
| **Domain 1: Research team and reflexivity** |  |  |
| Personal Characteristics |  |  |
| 1. | Interviewer/facilitator | Which author/s conducted the interview or focus group? Page 7 |
| 1. | Credentials | What were the researcher's credentials? *E.g. PhD, MD* Page 7 |
| 2. | Occupation | What was their occupation at the time of the study? Page 7 |
| 3. | Gender | Was the researcher male or female? Page 7 |
| 5. | Experience and training | What experience or training did the researcher have? Page 7 |
| Relationship with participants |  |  |
| 6. | Relationship established | Was a relationship established prior to study commencement? Page 6 |
| 7. | Participant knowledge of the interviewer | What did the participants know about the researcher? e*.g. personal goals, reasons for doing the research* OSM Figures S1 and S1 |
| 8. | Interviewer characteristics | What characteristics were reported about the interviewer/facilitator? e.g. *Bias, assumptions, reasons and interests in the research topic* Page 7 |
| **Domain 1: study design** |  |  |
| Theoretical framework |  |  |
| 9. | Methodological orientation and Theory | What methodological orientation was stated to underpin the study? *e.g. grounded theory, discourse analysis, ethnography, phenomenology, content analysis* Page 7 |
| Participant selection |  |  |
| 10. | Sampling | How were participants selected? *e.g. purposive, convenience, consecutive, snowball* Page 6 and 7 |
| 11. | Method of approach | How were participants approached? e*.g. face-to-face, telephone, mail, email* Page 6 and 7 |
| 11. | Sample size | How many participants were in the study? Page 9 |
| 12. | Non-participation | How many people refused to participate or dropped out? Reasons? OSM Figure S6 |
| Setting |  |  |
| 13. | Setting of data collection | Where was the data collected? e*.g. home, clinic, workplace* Page 7 |
| 15. | Presence of non-participants | Was anyone else present besides the participants and researchers? Page 7 |
| 16. | Description of sample | What are the important characteristics of the sample? *e.g. demographic data, date* Page 9 and Table one |
| Data collection |  |  |
| 17. | Interview guide | Were questions, prompts, guides provided by the authors? Was it pilot tested? Page 7, OSM Figures S2,3 and 5 |
| 18. | Repeat interviews | Were repeat interviews carried out? If yes, how many? Page 7 |
| 19. | Audio/visual recording | Did the research use audio or visual recording to collect the data? Page 8 |
| 10. | Field notes | Were field notes made during and/or after the interview or focus group? Page 7 |
| 11. | Duration | What was the duration of the interviews or focus group? Page 9 |
| 11. | Data saturation | Was data saturation discussed? Page 8 |
| 12. | Transcripts returned | Were transcripts returned to participants for comment and/or correction? Page 8 |
| **Domain 2: analysis and findings**z |  |  |
| Data analysis |  |  |
| 13. | Number of data coders | How many data coders coded the data? Page 8 |
| 15. | Description of the coding tree | Did authors provide a description of the coding tree? OSM Table S1 |
| 16. | Derivation of themes | Were themes identified in advance or derived from the data? Page 8 |
| 17. | Software | What software, if applicable, was used to manage the data? Page 8 |
| 18. | Participant checking | Did participants provide feedback on the findings? Page 8 |
| Reporting |  |  |
| 19. | Quotations presented | Were participant quotations presented to illustrate the themes / findings? Was each quotation identified? e*.g. participant number* Table 1 |
| 20. | Data and findings consistent | Was there consistency between the data presented and the findings? Table S1 Results |
| 21. | Clarity of major themes | Were major themes clearly presented in the findings? Results |
| 21. | Clarity of minor themes | Is there a description of diverse cases or discussion of minor themes? Results |

Table S2 Final Coding Index

| Initial themes | Initial categories | **Macro**  National level | **Meso**  Level of service provider e.g. CGL, Cranstoun, Turning Point | **Micro**  Level of individual service  HAHP | **Micro**  Level of individual service  HALP | **Micro**  Level of individual service  LAHP | **Micro**  Level of individual service  LALP |
| --- | --- | --- | --- | --- | --- | --- | --- |
| 1. **Who does and doesn’t receive specialist alcohol treatment** | 1.1 Characteristics of those receiving treatment |  |  |  |  |  |  |
|  | 1.2 Characteristics of those not receiving treatment |  |  |  |  |  |  |
|  | 1.3 Priority groups |  |  |  |  |  |  |
|  | 1.4 Excluded groups |  |  |  |  |  |  |
|  | 1.5 Complex needs |  |  |  |  |  |  |
|  | 1.6 Relationship and referral from with mental health services |  |  |  |  |  |  |
| 2. **Access to specialist alcohol treatment services** | 2.1 Barriers to access specialist alcohol services |  | | | | | |
|  | 2.1.1 Stigma associated with specialist alcohol services |  |  |  |  |  |  |
|  | 2.1.2 Stigma to individuals |  |  |  |  |  |  |
|  | 2.1.3 Transport |  |  |  |  |  |  |
|  | 2.1.4 Location of service |  |  |  |  |  |  |
|  | 2.1.5 Co-location with drug services |  |  |  |  |  |  |
|  | 2.1.6 Personal barriers |  |  |  |  |  |  |
|  | 2.1.7 Other |  |  |  |  |  |  |
|  | 2.2 Facilitators to access specialist alcohol services |  |  |  |  |  |  |
|  | 2.3 Innovation and creativity |  |  |  |  |  |  |
|  | 2.4 Funding cuts |  |  |  |  |  |  |
|  | 2.5 Recommissioning |  |  |  |  |  |  |
|  | 2.6 Marketing and promotion of specialist alcohol service |  |  |  |  |  |  |
| 3. **Hospitalisation** | 3.1 Experiences of hospitalisation due to alcohol |  |  |  |  |  |  |
|  | 3.2 Characteristics of hospital attendees |  |  |  |  |  |  |
|  | 3.3 Trends in hospitalisation |  |  |  |  |  |  |
|  | 3.4 Relationship of services with hospital |  |  |  |  |  |  |
